# Genomic testing in gamete donors: clinicians’ perspectives on recontact and pre-donation genetic counseling

**DOI:** 10.1007/s10815-025-03694-0

**Published:** 2025-10-25

**Authors:** Yulia Kaplan Idelchuk, Maya Sabatello, Hagit Hochner, Shiri Shkedi Rafid

**Affiliations:** 1 Faculty of Medicine, Hebrew University of Jerusalem, Jerusalem, Israel; 2 Department of Medicine, Columbia University, New York, NY USA; 3 Department of Medical Humanities and Ethics, Columbia University, New York, NY USA; 4 Braun School of Public Health, Hebrew University of Jerusalem, Jerusalem, Israel; 5 Department of Genetics, Hadassah Medical Center and Faculty of Medicine, Hebrew University of Jerusalem, Jerusalem, Israel

**Keywords:** Gamete donation, Genomic testing, Recontact, Qualitative research, Genetic counseling

## Abstract

**Purpose:**

Widespread use of genome-wide testing during pregnancy and throughout life raises clinical, legal, and ethical questions. Results from such tests in the context of gamete donation have implications also for the donor and other recipients. Current guidelines do not fully address this matter. We aim to provide empirical data on clinician’s perspectives towards pre-donation genetic counseling and recontacting sperm donors for additional genome-wide genetic testing.

**Methods:**

In-depth interviews were conducted with 19 healthcare professionals across Israel from different disciplines (sperm bank directors, fertility and genetic specialists) and were analyzed using the grounded theory approach.

**Results:**

Overall participants emphasized the importance of pre-donation comprehensive genetic counseling, which should cover future potential genetic tests findings, and their implications for both donor and offspring health, along with offering donors the choice to allow recontact later on. Approximately half of the participants believed recontacting donors should happen before performing broader genetic tests on the offspring, mainly due to possible implications for the donor’s health. In contrast, about a third of participants advocated recontacting donors only if clinically significant findings are identified, driven by practical reasons concerning the benefit to the offspring and time-sensitive situations, like pregnancy.

**Conclusion:**

This study highlights the need for clear guidelines regarding donor recontact in the context of expanding genetic testing. A strong consensus exists on the necessity of comprehensive pre-donation genetic counseling, divergent views on recontact emphasize the need to balance the implications for donor health with practical considerations for offspring and timely medical interventions.

**Supplementary Information:**

The online version contains supplementary material available at 10.1007/s10815-025-03694-0.

## Introduction

Recently, a case of a sperm donor who passed on a faulty cancer predisposition gene came to light in Europe [1]. The donor, in good health himself, donated through the European Sperm Bank between 2008 and 2015. Additional genetic testing was issued in the donor’s sperm after two families independently contacted their fertility clinics as their children developed cancers that appeared to be linked to a rare genetic variant in the *TP53* gene. This variant was not classified as pathogenic at the time of donation, and standard genetic testing done prior to donation could not detect such variants. Dozens of children, belonging to over 40 families, from 6 European countries, have so far tested for the *TP53* variant, many of whom found carriers and can now benefit from strict surveillance for the early detection of cancer. This case illustrates challenges that could arise in gamete donation, especially when gametes are shipped between countries, and over many years.

In the era of widespread genome-wide testing during pregnancy and throughout life, clinical, legal, and ethical questions arise, regarding the breadth of genomic tests carried out prior to gamete donation, recontacting donors for genetic testing or disclosure, and consent to genetic testing and recontact.

Donor conception accounts for 1 in 170 births and nearly 1 in 6 IVF births in the UK. UK births from donor sperm tripled over the past decade, driven by single and same-sex parents [2]. Similar trends are seen in the USA [3] and Israel [4], with global sperm bank market projections reaching USD 8 billion by 2030 [5].

The rapid development of medical genetics may create a gap between donor testing at donation and later testing on donor-conceived individuals. Donors are typically screened for limited autosomal recessive conditions. However, broader testing during pregnancy or on offspring may identify complex findings, including uncertain results, findings with incomplete penetrance and/or varied age at-onset [6]. Trio testing (comparing offspring to biological parents) significantly aids in reaching a diagnosis in cases of uncertain variants identified in an offspring [6, 7]. Additionally, pathogenic findings in donor-conceived individuals can have health implications for the donor and other offspring (such as the *TP53* case) [8], yet donor testing may be hindered by geographic distance, lack of commitment, or loss of follow-up. One example of challenging genetic findings are secondary findings, i.e., genetic results unrelated to the primary reason for testing that may have important health implications. The American College of Medical Genetics and Genomics (ACMG) recommends the deliberate analysis and reporting of a specific list of genes (currently 81) associated with medically actionable conditions [9]. These findings can reveal predispositions to conditions such as hereditary cancers or cardiovascular diseases, offering opportunities for early intervention. However, they also raise ethical and practical challenges related to informed consent, patient preferences, and the management of unexpected risk information.

Current guidelines in the USA [10], Europe [11], and Israel [12, 13] not sufficiently address these issues. The American Society for Reproductive Medicine (ASRM) guidelines focus on carrier screening for autosomal recessive conditions. The European Society of Human Reproduction and Embryology (ESHRE) advises against advanced genomic testing as exome or genome sequencing in donors due to validity concerns and offers no guidance for managing findings in offspring. Israeli Ministry of Health (MOH) guidelines for sperm [12] (2007) and egg [13] (2011) donations recommend only Tay-Sachs and karyotype screening for sperm donors and Fragile X testing for egg donors, reflecting outdated standards. Nevertheless, Israeli sperm donors are required to sign a consent form stating their DNA might be used in future testing. However, this is not common practice in different sperm banks worldwide.

In more detail, under the current Israeli MOH guidelines, both sperm and oocyte donation must remain anonymous, with no access to identifying information about either recipients or offspring. Eligible donors are single men aged 18–30 or women aged 21–35, who undergo screening for infectious diseases (HIV, HBsAg, HCV, syphilis). While the requirements for anonymity and infectious diseases testing are similar for both, important differences exist. For example, since egg donation involves greater medical risks, each donor may contribute to a maximum of three recipients, whereas no explicit limit is placed on the number of recipients for sperm donation. However, neither set of regulations addresses more complex scenarios, such as the death of a donor. In such cases, sperm banks handle the situation on a case-by-case basis.

Moreover, notable differences exist among the major religions in Israel, i.e., Judaism, Christianity, and Islam, with respect to their perspectives on gamete donation.

In Sunni Islam, gamete donation of any kind is forbidden, based on the principle that no third party should intrude upon the marital functions of sex and procreation. In contrast, Shi’a Islamic law permits both sperm and egg donation [14]. In Jewish Halacha, sperm and egg donation are generally discouraged due to concerns such as the risk of inadvertent incest and the difficulty of determining parenthood. Nevertheless, Halachic authorities acknowledge that in cases where no other option for having children exists, both sperm and egg donations may be permitted [15]. In the main Christian denominations, Catholicism and Protestantism, gamete donation is prohibited [14]. It is noteworthy that these religious restrictions apply mainly to religiously observant individuals and less so to the broader secular population. As aforementioned, regulation in Israel allows both types of gamete donation.

ASRM states that pre-test genetic counseling for donors should discuss future recontact for additional samples/testing and potential health risks. Israeli 2007 MOH guidelines allow broad donor consent for future DNA use to improve child health, without recontacting donors for their consent. However, these guidelines reflect the genetic testing standards of that time and do not account for the significant advancements in genomic testing technologies since then. Moreover, MOH guidelines only apply to Israeli donors, while international donors (US, Denmark) are increasingly used in Israel and Europe [2], creating regulatory gaps.

Previous empirical research on gamete donation focused on donor motivations to donate, and anonymity vs. identity disclosure [16]. Limited data exists on clinicians’ experiences and attitudes toward integrating genomic medicine into gamete donation. We conducted a study to explore clinicians’ views on genetic testing in gamete donation, identify challenges, and propose solutions. We focused on sperm donation due to its higher prevalence in Israel compared to oocyte donation.

## Methods

### Study population

We recruited 19 healthcare professionals (HCPs) from 13 medical centers across all major Israeli regions using professional networks and snowball sampling. All approached professionals agreed to participate. Inclusion required at least 5 years of clinical experience in relevant fields.

Participants included six sperm bank directors, four in vitro fertilization (IVF) specialists, five genetic counselors (GCs), and four medical geneticists (MGs). All MGs and sperm bank directors had prior training/practice in Obstetrics & Gynecology. Ethical approval was obtained from Hadassah Medical Center’s ethics committee [0058‐22‐HMO].

### Data collection and analysis

Semi-structured, in-depth interviews were conducted from April 2022 to April 2024 by Y.K. (ten face-to-face, one by video, eight by phone). Interviews averaged 40 min were audio-recorded and transcribed verbatim. The interview guide, developed based on our team’s clinical experience, covered questions regarding pre-donation genetic tests, informed consent, recontacting donors, and disclosure policy. An English translation of a Hebrew interview guide is provided as supplementary information. This paper focuses on pre-donation counseling and recontact for additional testing.

Data analysis followed grounded theory approach [17]. Two researchers (Y.K. and S.S.R.) independently coded transcripts line-by-line, categorizing until key themes emerged. New findings were discussed collaboratively by the research team for consensus, enhancing validity. Representative quotes (translated from Hebrew) illustrate themes.

## Results

Table 1 details participant characteristics.

**Table 1:** Participants’ characteristics

Characteristics	*n* (%)
*Professional background*	
Sperm bank director	6 (32)
Genetic counselor (GC)	5 (26)
Medical Geneticist^a^(MG)	4 (21)
IVF specialists	4 (21)
*Gender*	
Male	7 (37)
Female	12 (63)
*Geographic area*	
North	2 (10)
Center	8 (42)
Jerusalem	4 (22)
South	5 (26)
*Years of experience*^*a,b*^	
5–10	4 (21)
11–15	4 (21)
> 15	11 (58)

^a^In Israel, medical geneticists first specialize in other fields. All the medical geneticists interviewed in this study were initially trained in and practiced Obstetrics & Gynecology. The years of experience reflect their experience as medical geneticists

^b^In Israel all sperm bank directors are trained in and practice Obstetrics & Gynecology and particularly IVF

Main themes regarding recontacting donors prior to new genetic tests are described below.

### Recontact donors prior to performing new genetic tests on donors’ DNA is the right thing to do

Approximately half of participants (four MGs, four sperm bank directors, one IVF specialist) believed recontacting sperm donors is ideal when planning a broader genetic test (such as exome sequencing). This requires informing the donor about the test, its results, and implications. Recontact also requires his initial consent.

If we break the ‘contract’ and suddenly introduce a new kind of test, such as exome sequencing, of which the donor was not initially informed, we will need to go back to him. At the time of donation, the donor needs to be informed and give his consent [to being recontacted]. We can’t just surprise him with a new kind of test… If it’s a completely new kind of test with potential implications for his own health, we should inform him and obtain his consent once again. (P13, GC, Female).

Two main arguments supported this view:

- Clinicians anticipated difficulties informing donors about positive, health-implicating genetic results without further preparation:

He needs to be informed of all these things [possible test results] beforehand. For example, if he is unexpectedly identified as a *BRCA* carrier, does he want to know? These are things he needs to be aware of in advance, rather than discovering them only after undergoing exome sequencing, which would create a significant problem. (P7, IVF specialist, Female)'

(b) Donors’ opinions can change over time.

It is sensible to assume that people’s opinions can change over time. If someone agreed to something at age 20, he may view it differently by age 30. Therefore, it might not be fair to rely on consent given in a very specific context. (P1, GC, Female)

Conversely, two participants (one sperm bank director, one MG) felt recontact shouldn’t be obligatory if testing is crucial for offspring health.

I would rather inform him online that I will be conducting a comprehensive test. However, if that’s not possible, I don’t want this to hold me back. (P14, MG, Female).

This highlights the tension between donor autonomy and offspring well-being. A genetic counselor described the dilemma:

When you go back to someone with new information, you can’t be sure if they appreciate being informed, or if they feel it is an intrusion into their privacy and daily life. Even if you believe you are doing good and have good intentions, these are indeed challenging and thought-provoking questions. (P15, GC, Female).

## Recontact should only be done post-testing, if major findings are identified

About a third of participants (two sperm bank directors, three genetic health professionals, one IVF specialist) advocated recontacting donors only if pathogenic or clinically significant findings are identified, not before testing. The main argument was practical: searching for former donors is time-consuming, and most expanded tests yield normal results.

Recipient and offspring well-being was another consideration, especially in urgent pregnancy situations where delays from locating donors might be critical:

I think it would be very distressing for the recipient if I had to tell her, ‘Listen, I can’t find him, he’s not answering the phone, and I don’t know what to do.’ (P14, MG, Female).

Some participants also considered donors’ well-being; recontacting long after donation, without significant findings, could be disruptive:

Many donors provide their samples and move on with their lives, and they may not want to hear from us. It’s important to respect their wishes. (P9, MG, Male).

Two sperm bank directors shared that donors generally favor comprehensive genetic testing, partly for personal health benefits. Based on donors’ favorable attitude toward additional genetic information, pre-testing recontact is not mandatory.

We added a statement [to the consent form]: ‘I wish/do not wish to receive results relevant to my health or the health of my descendants.’ We explain that if something related to their health is found, they will be notified. Most have requested to receive such results. One individual initially chose not to, but later called back to say he had reconsidered and decided he did want to receive such results. (P3, Sperm bank director, Female).

Most participants agreed donors aren’t interested in recontact for every new test:

They [the donors] have signed a consent form, and their DNA sample is stored for several years. If we choose to conduct additional tests, we don’t need to seek their permission each time. I believe they themselves don’t want it [to be recontacted before further testing]. (P13, GC, Female).

Obtaining broad, comprehensive consent at initial donation could resolve recontact difficulties:

In terms of service to the women, it is clearly much better [to obtain broad consent prior to donation], especially if the donor is from abroad and receiving his cooperation may be difficult. (P15, GC, Female).

[signing a broad consent] is the easiest thing for sperm bank directors, the easiest for the system. (P7, IVF specialist, Female).

If broad consent at the time of donation becomes standard practice—whether due to practical considerations or for the well-being of both the donor and recipient—participants acknowledged a need for much more detailed pre-donation genetic counselling than what is currently practiced.

## Pre-donation genetic counseling is highly desired

Almost all participants, especially genetic health professionals, emphasized the importance of comprehensive genetic counseling prior to donation. Six genetic health professionals, three sperm bank directors, and one IVF specialist noted counseling should extend beyond obtaining family history to include detailed explanations of future possible genetic tests, types of findings, and implications for donor and offspring health. Exome sequencing was frequently cited as an example of a genetic test that may have such implications.

In an ideal world, I believe that [pre-donation] counselling should be similar to what is provided before diagnostic genetic testing, rather than just focusing on tests mostly relevant to offspring health. It’s not just about taking a family history, but about explaining what is involved in an exome test and what kind of findings could be revealed. (P15, GC, Female).

Most participants recognized genetic counseling should ideally be provided by a genetics specialist.

What do we as gynecologists understand? Probably not genetics. (P5, Sperm bank director, Male).

I don’t trust that a gynecologist will thoroughly explain what a broad genetic test is. I genuinely believe that a genetic health professional should provide such an explanation. (P14, MG, Female).

However, due to shortages of genetic health professionals, participants acknowledged that non-genetics clinicians could provide counseling with appropriate training:

In principle, a well-trained sperm bank director, with proper guidance from a genetic health professional, can effectively explain genetic testing to donors. (P19, GC, Female).

The question is whether a gynecologist’s explanation about exome testing for dominant conditions is sufficient? I assume it might be, but not because it’s optimal—rather because it’s the system’s limitations. (P13, GC, Female).

Some participants also suggested that during counseling, donors could choose what information they wish to receive and the possibility of recontacting them prior to performing future genetic tests.

## Involving donors in recontact decisions prior to donation

A possible compromise, between always recontacting donors for additional testing and contacting them only if pathogenic variants are identified, as suggested by eleven participants (six genetic health professionals and five fertility experts including three sperm bank directors), is to ask donors, at the time of donation, which course of action they would prefer:

Maybe we should have them sign a consent covering all potential possibilities, such as whether they agree to future expansions of tests. I assume that most will agree. Additionally, we could include a choice on whether they want to be informed about the results. They could opt for either: ‘No, I don’t want to, I’m just donating and moving on’ or ‘Yes.’ (P6, MG, Female).

Nevertheless, three participants (two genetic health professionals, one sperm bank director) argued that donor attitude toward future testing should be a selection parameter. Donors refusing recontact or genetic information might not be suitable.

If someone says he doesn’t want to learn about these kind of things [adult- onset conditions] about himself, perhaps he is not suitable to become a donor…. Flexibility is essential for donation. Donors need to be open to advanced technologies that we can no longer ignore. Unlike in the past, when we only performed karyotyping, today’s advanced tests reveal various findings, and testing the parents is important for interpretation. If a donor is unwilling to undergo these tests, it poses a problem, at least in my view. It raises the question of whether we should accept their donation under such circumstances. (P19, GC, Female).

This aligns with two genetic health professionals’ views that recipient needs outweigh donor rights:

The motivation for donation is not for the benefit of the donor. The word ‘donation’ implies something a person gives willingly. Even if the donor receives compensation for their time or something similar, priority should be given to the recipients’ needs. Therefore, everything should be done to meet their needs, including those that may arise during pregnancy. (P9, MG, male).

Alternatively, if donors who are not available for further genetic testing are still recruited, recipients should know this in advance:

On each sperm donation they are selling to women they should know upfront that this sperm donor doesn’t have stored DNA, the donor didn’t consent for recontact, he doesn’t want to know, gave his sperm and that’s it. All these things should be communicated. (P7, IVF Specialist, Female).

Some participants suggested genetic counseling for recipients to explain donor availability implications for testing:

The purpose of counseling for the recipient is to clarify the limitations of genetic testing in donor conceived pregnancies. It’s not always possible to decipher everything. That’s why I believe that preliminary counseling and understanding of what can and cannot be done, along with setting expectations, are extremely important. It’s essential to understand that when you conceive with a donor, you may face certain challenges that are greater than in other situations. (P1, GC, Female).

## Discussion

The era of advanced genomic testing introduces challenges to users and providers alike. Gamete donation may complicate things even further, given the elapse in time and location between the initial donation and genetic testing carried out on offspring conceived with the assistance of gamete donation. Uncertain and secondary findings in offspring often necessitate donor recontact. Uncertain findings can reach 30% in exome sequencing, requiring parental information for interpretation [18]. Secondary findings are identified at about 2.5% of adults [19]. These prevalence rates highlight the critical need for clear, regulated recontact instructions. One practical solution is to store DNA from each donor worldwide for potential future genetic testing. Yet this approach doesn’t resolve the dilemmas addressed in our paper, such as whether donors should be recontacted before performing additional genetic tests on their samples.

Our study participants generally aligned with ASRM [10] guidelines, supporting donor recontact before additional genetic tests. However, they acknowledged situations where recontact might be unnecessary or impractical.

One argument for recontact was the possibility of donors changing their preferences over time. Horton et al. suggests consent is most effective for binary, time-locked choices [20]. In donation, donors need to process a lot of information prior to donation, which potentially prevents them from deep reflection on long-term genetic testing consequences, thus initial consent might not reflect evolving preferences.

Conversely, recontacting donors for every additional genetic test is impractical. A nuanced approach is needed: recontact only for expanded tests with significant implications for the donor’s own health (e.g., exome or genome sequencing), but not for similar tests (e.g., additional carrier screening for recessive conditions).

Several studies show people generally prefer comprehensive genetic information for themselves and their offspring, including adult-onset diseases [21–23]. Pennings et al. found that over 80% of sperm donors desired to receive all results from additional genetic tests [24]. This may favor recontacting only those donors who explicitly requested it, or only in cases of a pathogenic result.

It should be noted that recontact should be reciprocal, meaning that donors are requested to inform sperm banks about illness in themselves or their family members, and sperm banks should then inform recipients for the sake of their offspring. However, this requires mechanisms for maintaining up to date recipient details, and it is practically impossible to convey this information directly to the offspring.

Various consent models could be implemented into the field of gamete donation: Dynamic consent, using personalized online platforms, could facilitate ongoing, two-way interaction between clinicians and donors for updates and new consent [25]. Challenges of this approach are mainly technical: robust infrastructure, costs, and donor engagement. Meta-consent [26–28] allows donors to specify preferences for different test types (e.g., broad consent for carrier screening, specific consent for exome sequencing). Critics argue meta-consent doesn't truly meet the donor’s actual needs, since at time of consent the donor is unable to consent to an unknown future event, making the consent essentially meaningless [29].

Regardless of the consent model, our findings emphasize the need for comprehensive genetic counseling for gamete donors. Traditionally, counseling focused on obtaining family history and explaining the results of screening for autosomal recessive conditions. With genomic medicine, pre-donation genetic counseling must expand to include potential future tests, detected genetic variations, and their implications for donor and offspring health. Counseling should also explicitly address potential recontact circumstances.

Some participants suggested allowing donors to choose whether they wish to be recontacted and undergo additional testing. This could enhance donor engagement and reduce logistical issues. However, it might not resolve opinion changes over time and could create ethical dilemmas. For example, if a life-saving secondary finding emerges in an opted-out donor, disclosure questions arise. While ACMG permits individuals to opt out of secondary findings in clinical settings [30], in gamete donation, such information could be derived from multiple donor-conceived children, leading to an almost certain diagnosis in the donor, regardless of their initial preference (once again, illustrated in the aforementioned *TP53* case).

One controversial solution raised by a few participants was to exclude donors objecting to future testing/recontact. This “punitive” approach raises ethical concerns, potentially violating the right not to know one’s genetic makeup and pressure donors to agree to receiving genetic information on themselves even if unwarranted, only to ensure their acceptance as donors. Mukherjee et al. note donor interests are often overlooked because recipients bear costs and their interests are prioritized [31]. Moreover, introducing varied levels of genetic information on donors may create inequity, as gametes obtained from donors available for additional information may be more expensive.

Given genomic complexity, participants supported involving genetic health professionals in pre-donation counseling. However, specialist shortages might make this unfeasible [32]. A practical approach involves training non-genetics clinicians to obtain consent, supplemented by educational virtual tools.

Recipient parents must be informed about the potential “gap” between donor genetic information at donation and future insights from offspring testing. The challenges of recontacting donors and obtaining samples/consent should be communicated pre-donation, as this may impact recipients’ donor choice.

In summary, we propose that genetic testing in the gamete donation process should include the following:

- Comprehensive pre-donation genetic counseling for both donors and recipients: covering family history, current/future genetic tests, evolving genetic tests including those not yet foreseeable, potential future recontact circumstances, and attitudes toward different genetic findings (e.g., incomplete penetrance, adult-onset, recessive vs. dominant variants). If donors choose disclosure preferences, this must be documented and available to recipients. Regulation should ensure equity, prevent pressure on opting-out donors and higher costs for opting-in donors.
- Specialized training if counseling is done by clinicians outside of genetics, specific training is recommended.
- Development of a dynamic communication platform to facilitate ongoing, bidirectional, and user-friendly contact between donors and fertility clinics, enabling updates and future recontact when necessary. Until such a platform is developed, donors should be asked to provide an active email address and to keep it active for the purpose of possible future notifications.

### Limitations

This study focused on sperm donations, being most common in Israel; similar suggestions may be relevant for egg donation; however, further empirical validation is required.

Responder bias is a potential limitation due to professional acquaintance with the interviewer. Guaranteed anonymity and neutral questions were used to minimize this.

Participants primarily work in the public sector; private sector opinions may differ.

Lastly, Israel’s high availability and uptake of prenatal genetic testing might have influenced our findings, potentially limiting generalizability to other countries.

## Conclusions

Taking together the increasing prevalence of donor-conceived families, rapid genomic advancements, international gamete sharing and geographic distances, additional genetic testing and recontact must be regulated, ideally internationally, and transparent to both donors and recipients. Additional empirical studies should be conducted on the donors’ and recipients’ perspectives.

## Supplementary Information

Below is the link to the electronic supplementary material.

ESM 1 (DOCX 22.9 KB)

## Data Availability

Data can be accessed upon request.
