# Supplementary material for: Genomic testing in gamete donors: clinicians’ perspectives on recontact and pre-donation genetic counseling
Source: J Assist Reprod Genet. 2025 Oct 25;43(1):109–16. doi: 10.1007/s10815-025-03694-0 (PMC12831762; doi:10.1007/s10815-025-03694-0)
Supplement: Supplementary file 1 — (DOCX 22.9 KB) [file 10815_2025_3694_MOESM1_ESM.docx]

**Sperm Bank directors Interview Guide**

**Donor Recruitment Process:**

- What process does each potential donor go through?
- What proportion of donors in your bank are Israeli? And how many are foreign?
- What genetic tests are performed on Israeli donors, and which are performed on foreign donors?
- Is there a difference in the genetic screening process between different donors? If so, are there criteria for referrals to specific tests? How were these criteria established?
- What genetic tests should be performed on women seeking donor sperm?
- In your experience, does the extent of genetic screening undergone by a donor affect recipient choice?
- To what extent do you consider the donor in the context of genetic evaluation before donation?
- What explanation does the donor receive about the genetic testing and possible results? Who provides the explanation?
- Do you think every recipient should undergo genetic counseling? If so, for what purpose?
- What type of informed consent do donors (both Israeli and foreign) sign? Does it include consent for future genetic testing of their DNA? Can you share a sample consent form?
- What explanation is given regarding this part? Does it include details about possible tests to be done (by recipients or during pregnancy/post-birth), and their implications? Does it address the possibility of findings of uncertain significance? Findings suggest an increased risk of late-onset diseases (e.g., cancer)?
- What responses have you encountered to these explanations?
- During donor recruitment, is there reference to the possibility of recontacting the donor in the future if genetic findings arise in recipients or offspring? Are donors given the option to allow or decline such future contact?
- Have you encountered complex cases relating to genetic evaluation during donor selection?

**If Advanced Genetic Testing is Performed on the Donor (e.g., CMA, Exome):**

- Which findings, in your opinion, are worthy of reporting? Should carrier status for recessive conditions be reported? What about findings indicating increased risk for late-onset diseases (like cancer)? What about uncertain findings, such as a 20% risk for neurodevelopmental disorders (e.g., autism)?
- Which findings, if any, should disqualify a donor?

**If Significant Genetic Findings Are Identified in a Fetus or Child Conceived Using Donor Sperm** (including cases where a finding is discovered in the offspring, but the donor cannot be tested, such as with foreign donors):

- Under what circumstances, in your opinion, should other recipients who used the same donor be contacted? (Childhood/adulthood onset, only pathogenic findings/also uncertain significance, full/partial penetrance, severity, de novo...)
- Under what circumstances, in your opinion, should the findings be reported to the MOH/ other sperm banks (in case of a foreign donor)
- Under what circumstances, in your opinion, should the use of the donor’s sperm be discontinued? (Same categories)
- Should the donor be tested? What kind of findings, in your opinion, are worthy of reporting? (e.g., childhood-onset conditions, adult-onset diseases, findings with certain penetrance or severity)
- How should one proceed if the donor cannot be tested?
  *Ensure the interview also covers both inherited and de novo findings in the embryo/child that are not present in the donor.*

Advanced testing may sometimes reveal variants of uncertain clinical significance. If such a variant is found in a fetus/child and not in the mother, testing the donor may be crucial in determining whether the variant is relevant or likely unrelated (e.g., inherited from a healthy donor).

- What is your position on testing donors for such findings when they are discovered?
- Should the donor be informed that testing is being done?
- Do you follow any specific guidelines?

**If Genetic Test Results Are Reported to the Donor and/or Other Recipients:**

- Who decides which results to report?
- How are the results delivered (in-person, by phone, in writing)? By whom?
- What kinds of reactions have you encountered? (Especially regarding findings of uncertain significance, carrier status for recessive diseases, or genes associated with adult-onset conditions)
- As a Health care professional - What parts of the donor’s genetic screening process (preconception and during pregnancy) do you feel comfortable with? Which aspects make you uncomfortable?
- Would you prefer national-level guidelines, or do you think such decisions should remain with the clinical team?
- Is there anything in the current process you believe should be changed?

**If Unclear Genetic Results Are Obtained:**

- Is there follow-up on new scientific developments, and are donors/recipients/patients contacted if the significance of the finding becomes clearer?

**General Questions:**

- How many years have you been working in this field?
- How would you describe yourself: secular; traditional; religious; ultra-Orthodox?
- Gender?
